# The Employment of Telegraph Sutures in Closing Wounds in Various Cases

**Published:** 1863-04

**Authors:** 


### THE EMPLOYMENT OF TELEGRAPH SUTURES IN CLOSING WOUNDS IN VARIOUS CASES.

[Under the care of Mr. Erichsen.]

Mr. Clover has introduced a new form of suture, which appears to possess many advantages over those ordinarily employed, whether of silk thread, or metal. It consists in the adoption of a very fine copper wire covered with gutta-percha—in fact, a minute telegraph wire, and therefore receiving the name of "telegraph sutures." We have seen them used in a number of cases at University College Hospital, wherein Mr. Clover was himself allowed to apply them, because, as we heard Mr. Erichsen remark, it was but fair to allow the inventor to show the application of his own invention.

One of the fisrt cases which came under our notice was one in which Mr. Erichsen removed a tumor the size of an orange from the right parotid region of an elderly man on the 22d of October last. The growth, although movable, was situated in a region requiring care to exercise it, for it lay between the angle of the jaw and mastoid process, and did not dip behind the former. The tumor proved rather hard on making a section, and was fibro-nucleated in character—a form that is considered to be somewhat rare. The incision used was ⊥ shaped, and this was closed with a fine telegraph wire, in the coi tinuous or Clover's suture, by Mr. Clover; a small portion being left only to permit of the drain of secretion.

On the 17th inst. the edges of a double hare-lip in an infant were pared by Mr. Erichsen, and evenly brought together by deep, interrupted telegraph sutures by Mr. Clover. These, the former gentleman remarked, leave no scar--nothing at all like the hare-lip pin. He further stated that he had used them on the face of a gentleman from whom he had removed a tumor ten days previously, and no cicatrix was visible. He had also employed them in other cases. The silver wire, he observed, has the disadvantage of being very rigid, and does not tie well. The telegraph wire, on the contrary, is quite soft, and forms a knot like ordinary silk thread; it can be cut like silk, and be as readily taken out; it can, moreover, be used with a fine sewing needle.—*London Lancet.*

